# Shift Work and Early Arterial Stiffness: A Systematic Review

**DOI:** 10.3390/ijerph192114569

**Published:** 2022-11-06

**Authors:** Waléria D. P. Gusmão, Isabele R. O. M. Pureza, Claudia R. C. Moreno

**Affiliations:** 1 Department of Health, Life Cycles and Society, School of Public Health, University of Sao Paulo, Sao Paulo 01246-904, Brazil; 2 Department of Nutrition, Campus I—Prof. Eduardo Almeida, Centro Universitário Cesmac, Maceió 57051-160, Brazil

**Keywords:** shift work, night work, arteriosclerosis

## Abstract

Shift work is a way of organizing rotating schedules throughout the day. This can include 1–3 shifts for the same person on a rotational basis with other workers. Schedules that include night work have been associated with cardiovascular risk, mainly due to circadian misalignment. This systematic review sought to determine whether shift work is a risk factor for increased arterial stiffness. A systematic review of different databases was performed, using the following keywords: work shift, night work, arteriosclerosis, vascular stiffness, arterial stiffness, pulse wave velocity, and their Medical Subject Headings. We selected and analyzed 11 articles regarding pulse wave velocity as an indicator of arterial stiffness. Two studies identified higher levels of arterial stiffness in shift workers compared to day workers, while two studies found the opposite. In addition, four studies found no differences in arterial stiffness between shifts, two studies associated shorter sleep duration with arterial stiffness, and one study observed that physical activity could prevent adverse cardiovascular outcomes in shift workers. The findings are heterogeneous and preclude any robust conclusions. However, the present review points to the need for further studies to investigate arterial stiffness in shift workers, with greater control for confounding factors and longitudinal design.

## 1. Introduction

Shift work is an organizational form of working time, established to maintain the uninterrupted operation of workplaces through workers engaging in work schedules on a rotational basis [1,2]. Shift work is not a recent form of work organization, having existed since antiquity. This is characterized by work activity in shifts with a rotating pattern, clockwise or counterclockwise. It can involve shifts that start very early in the morning and may include part of the night or the whole night [3]. Worldwide production on a large scale and a practically uninterrupted, 24/7 basis, has led to the expansion of working hours beyond daytime hours, making shift work schedules increasingly common. Shift work can take the form of a rotating system incorporating three shifts (morning, afternoon, and night) on every day of the week, including weekends [4]. Workers engaged in shift work that includes night work have altered exposure to natural light and dark, which misaligns circadian rhythms [1]. Thus, night shift work has adverse health effects, predisposing workers to the development of metabolic and mental disorders and non-communicable chronic diseases, especially cardiovascular diseases (CVD) [5,6].

CVDs are one of the leading causes of death in Western countries. In the young and economically active population, 10–20% of total deaths are due to CVD associated with work characteristics [7]. One of the most widely acknowledged mechanisms for cardiovascular disease in shift workers is psychological stress [8]. Stress resulting from circadian misalignment and inversion of wake and sleep schedules can promote hypersecretion of cortisol and catecholamines, triggering hemodynamic and cardiovascular disorders [9,10]. Another hypothesis for the high risk of CVD among shift workers is that the inversion of the sleep–wake cycle due to night work may cause circadian disruption in blood pressure control mechanisms [11].

Arterial stiffness is an early, independent marker of increased risk of CVD and all-cause mortality [12]. The study of arterial stiffness has been increasing in the cardiovascular field. Arteries tend to lose elasticity naturally with senescence (aging) or stiffen earlier from prolonged exposure to harmful conditions such as blood pressure, hyperglycemia, inflammation, and oxidative stress [13,14]. Arterial stiffness has been significantly associated with arteriosclerosis and future cardiovascular outcomes in the general population and in patients with various diseases, even with adjustments for multiple confounders regarding cardiovascular risk [15,16].

There are several methods to measure arterial stiffness, with pulse wave velocity being the gold standard for expressing this phenomenon. The oscillometric method, which is non-invasive and the most used in clinical practice, allows the evaluation of central blood pressure parameters, arterial stiffness, and vascular functionality simultaneously [17,18].

There are few studies that have analyzed arterial stiffness in shift workers. Some studies have shown an association of night work with vascular changes that are predictive of CVD. There was an observed greater thickness of the intima-media layer of carotid arteries in young night workers [19]. Vascular alterations have also been observed in individuals with long work hours [20]. A study that analyzed atherosclerotic risk, using measures of pulse wave velocity (PWV) in rotating shift workers showed that, regardless of the type of rotation, shift workers had more health problems and a higher heart rate than day workers [21]. The repercussions arising from shift work and long hours likely trigger arterial stiffness and might culminate in early vascular aging in young individuals. Thus, the aim of this systematic review was to summarize the results of studies investigating whether shift work predisposes individuals to arterial stiffness and consequently a higher risk of cardiovascular diseases.

## 2. Materials and Methods

This systematic review entailed a critical analysis of articles in the literature and was conducted in accordance with the guidelines of the Preferred Reporting Items for Systematic Reviews and Meta-analyses (PRISMA) statement. The systematic review study was registered according to the international prospective register of systematic reviews [22], receiving PROSPERO number CRD42022336082. The approval of the Research Ethics Committee was not required because the study was a systematic review.

### 2.1. Search Strategy

The PICO strategy (population, intervention, control, and outcomes) was used to guide the research question (Table 1). Based on the question: “Does shift work lead to early arterial stiffness?”, searches were initially carried out to define the Medical Subject Headings (MeSH) terms and to determine the inclusion and exclusion criteria, allowing a thorough search of the databases and analysis of articles, increasing the reliability of the study. 

### 2.2. Eligibility

The search was carried out on the Cochrane, Embase, PubMed, Scielo, and Scopus databases, using the MeSH terms and inclusion and exclusion criteria described in Table 2. The references listed in the articles included in the review were also analyzed to identify additional studies addressing the research question.

The terms selected for the search were organized as strings, according to the pattern of each database. The search was performed by two reviewers independently, who carefully read the title and abstract of the studies to identify potentially relevant articles that met the inclusion criteria established for this review: (1) quantitative articles related to shift work, including night shifts, and arterial stiffness; (2) original and research articles, longitudinal studies, cohort studies and case–control studies; and (3) available online with free access and as full text, regardless of publication date. Disagreements regarding the eligibility of articles were resolved by consulting a third researcher.

## 3. Results

**Figure 1:**
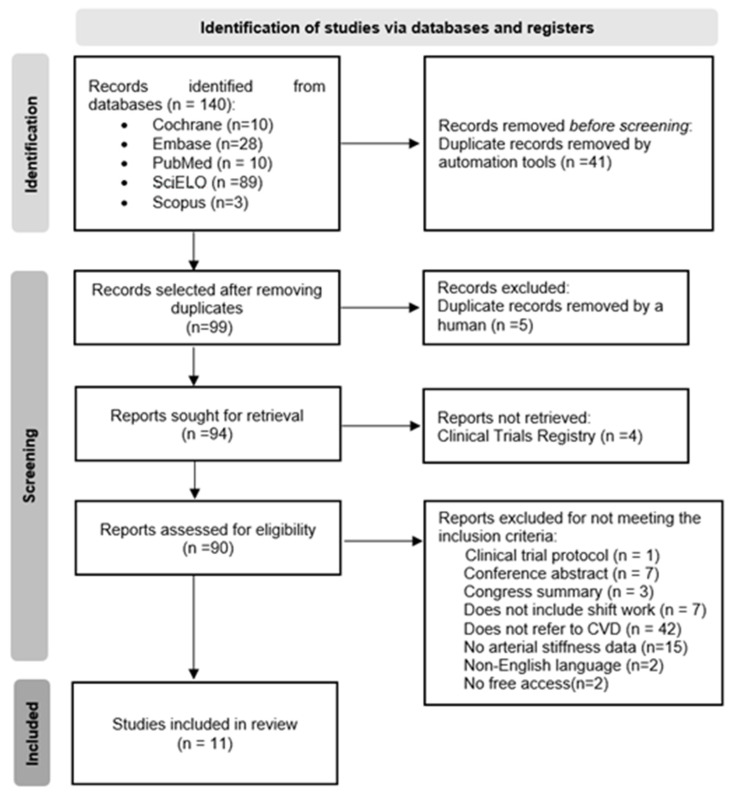
Flow diagram of process of search and selection of studies according to PRISMA criteria (Preferred Reporting Items for Systematics and Meta-Analyses).

A total of 140 articles were identified using the search strategy, comprising 10 on Cochrane, 28 on Embase, 10 on PubMed, 89 on Scielo, and 3 on Scopus databases. After the automated removal of duplicates (n = 41), 99 articles were selected for title and abstract screening. During the reading process, five duplicates and four records for clinical studies were detected and manually excluded. Of the remaining 90 articles, 79 were subsequently excluded for not meeting the inclusion criteria. The 11 remaining articles met the inclusion criteria and were selected. Therefore, after consensus between the two independent reviewers, 11 studies were included in this systematic review. A flow diagram of the study selection process based on PRISMA criteria is depicted in Figure 1.

This systematic review, including cross-sectional studies and cohort studies, revealed that there are only a few studies investigating shift work and arterial stiffness. The studies included in this review highlight that shift work can damage the physical and mental health of workers, and also increase the risk of cardiovascular disease.

**Figure 2:**
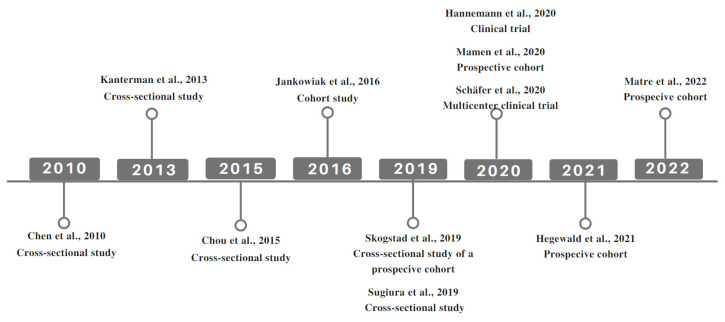
Timeline of publications and study types of articles included in this systematic review [21,23,24,25,26,27,28,29,30,31,32].

The present systematic review only included studies involving humans, since no simulated studies in animal models were found. The first article investigating the association of shift work and arterial stiffness was published in 2010 [23], possibly due to the wider access to non-invasive techniques for assessing arterial stiffness (Figure 2).

The characteristics of the selected articles are presented in Table 3, Table 4 and Table 5. In Table 3, data were tabulated by identifying the following variables: author and year of publication, country, study design, number of participants, gender and age, type of worker, exposure (type of shift work), and study duration or follow-up. Table 4 presents the objectives, collected data/methods, and assessment of arterial stiffness and groups. Finally, Table 5 describes the main results, confounder factors, and limitations of the studies.

Out of the eleven articles selected for this systematic review, five were cross-sectional studies, three were cohort studies, and three were interventional studies. Eight of the studies were carried out on the European continent and three on the Asian continent. The studies included adult healthy populations and number of participants varied greatly. The article with the fewest participants included 19 individuals [24] and the largest 10.475 people [25]. Eight articles had a predominance of male participants [21,23,24,25,26,27,29,31]. Females prevailed in the samples of three studies [28,30,32]. One study evaluated arterial stiffness in health professionals and observed that this indicator of arteriosclerosis did not differ statistically between men and women, despite the higher rate of work-life conflict in women [31].

The equipment used to assess pulse wave velocity (PWV) also varied widely. The methods used were: oscillometric [23,24,25,26,27,30,32] and waveform analysis [21,28,29,31]. The duration of the studies ranged from 8 weeks to 3 years. Considering PWV as an indicator of arterial stiffness, two articles identified higher PWV values in shift workers compared to day workers [23,25]. Some studies failed to identify any differences in arterial stiffness among workers on different shifts [21,27,30,32]. Conversely, two studies found higher PWV values in day workers than shift workers. Day workers who were older and had a higher BMI exhibited higher PWV values than night shift workers [26]. Another study observed significantly higher arterial stiffness values in day workers than in shift workers [29].

In the present review, two studies investigated the relationship between shorter sleep duration and arterial stiffness. In the study, short sleep (<6 h/day) and long working hours (>60 h/week) were non-linearly associated with arteriosclerosis [28]. However, another study observed a direct relationship between higher blood pressure levels and PWV related to short sleep episodes [24]. In a study evaluating the impact of physical activity as a preventive intervention against adverse cardiovascular outcomes in shift workers, was found that PWV values were negatively correlated with physical activity in both control and intervention groups [27].

In a study conducted to assess whether shift work, including long working hours and night shifts, was associated with the risk of developing CVD [26] it was observed that day workers had a higher PWV compared to shift workers. The results indicated that age and body mass index were positively associated with increased PWV, while a high level of physical activity had a protective effect on this indicator of arterial stiffness. Similarly, another study found that day workers had higher arterial stiffness rates than shift workers. Industrial workers on fixed day shifts had significantly higher levels of arterial stiffness when associated with smoking compared to shift workers [29].

Three of the studies included in this review observed no differences regarding arterial stiffness between shift workers compared to day workers. A study with metallurgists observed that PWV measurements were within the normal range and without statistically significant differences even when comparing shifts of fast rotation and clockwise direction with slow rotation and counterclockwise direction [21]. An intervention study involving physical exercise for eight weeks in industrial workers showed a reduction in blood pressure values in the intervention group [27].

The third study investigated the association between psychosocial stress and CVD risk in 576 medical staff in Taiwan [28]. PWV values were significantly higher in males, and after adjustment for gender, age, medical profession, working hours, type of work, depression, BMI, systolic and diastolic blood pressure, blood glucose, and cholesterol, only sleep duration < 6 h, and working hours > 60 h were significantly associated with an increased risk of atherosclerosis.

It is important to highlight the large differences regarding the shift schedules, professions, and workplaces in which the studies were performed.

**Table 3:** Characteristics, type of exposure and duration or follow-up of studies selected for inclusion in review.

Author and Year	Country	Study Design	N	Gender and Age	Type of Worker	Exposure	Study Duration/Follow-Up
Chen et al. (2010) [23]	Taiwan	Cross-sectional	184	100% males, mean age 42.2 years	Bus drivers	Night work on different days	3 months
Kantermann et al. (2013) [21]	Belgium	Cross-sectional	77	100% males, mean age 42 ± 7.6 years	Steel workers	Shift work, included night work	2009 to 2011
Chou et al. (2015) [28]	Taiwan	Cross-sectional	576	85.2% females, mean age 40.5 years	Health professionals	Shift work and night work	Not reported
Jankowiak et al. (2016) [25]	Germany	Cohort	10.475	74.2% males, age 35–64 years	Variety of professions	Current and previous night work	2007 to 2012
Skogstad et al. (2019) [26]	Norway	Cross-sectional prospective cohort	94	86.17% males, 49.7 years DW/40.3 years SW	Industrial employees	8 or 12 h night shift depending on the week	2018 to 2021
Sugiura et al. (2019) [29]	Japan	Cross-sectional	10.073	100% males, 46.6 ± 8.1 years	Industrial employees	Nighttime hours with or without an irregular schedule	2008 to 2009
Hannemann et al. (2020) [32]	Germany	Interventional	24	58% females/34.2 ± 8.6 years (IG), 75% females/37.3 ± 13.7 years (CG)	Not reported	At least 3 nightshifts/1 month for a period of at least 6 months.	24 weeks
Mamen et al. (2020) [27]	Norway	Interventional prospective cohort	94	86% males, 38.4 ± 11.5 CG/ 43.1 ± 10.5 IG years	Industrial employees	8 or 12 h night shift depending on the week	8 weeks
Schäfer et al. (2020) [30]	Austria and Germany	Interventional	64	79.9% female gender, 36.7 ± 12.2 years	Various professions	Shift work, included night work	24 weeks
Hegewald et al. (2021) [31]	Germany	Prospective cohort	2.426	51.1% male gender, 46.5 ± 7.3 years	Not reported	Shift work, included night work	2007 to 2012
Matre et al. (2022) [24]	Norway	Prospective cohort	19	89.47% male gender, 40.9 ± 11.5 years	Industrial employees	8 or 12 h night shift depending on the week	2018 to 2021, follow-up 5 weeks

N = number of participants, DW = day workers, SW = shift workers, IG = intervention group, CG = control group.

**Table 4:** Objective of the studies, collected data and methods, assessment of arterial stiffness, and groups analyzed.

Author and Year	Objective	Collected Data/Methods	Assessment of Arterial Stiffness	Groups
Chen et al. (2010) [23]	To examine the links between shift work and arteriosclerosis	Interview, measurement of BaPWV, BP, anthropometric data and laboratory assay	BaPWV (VP-2000—Colin Co., Ltd., Komaki, Japan)	EG = shift workers and CG = regular hours workers
Kantermann et al. (2013) [21]	To identify atherosclerotic risk in work shifts and to observe its relationship with social jetlag and shift schedule	Questionnaires, BMI, BP, AS measurements	PWV (Vicorder-Skidmore Medical, Bristol, UK)	Workers on fast CW and slow CC shifts
Chou et al. (2015) [28]	To explore the relationship between multiple work-related risk and arteriosclerosis	Eletronic questionnaire, Measurements of: arteriosclerosis, job stress, mental health and CVR (smoking status, BMI, FBG, CHOL and AH).	BaPWV/CAVI (VaSera VS-1000, Fukuda Denshi, Japan)	Analyzed according to: gender, job-related factors and presence of CVR
Jankowiak et al. (2016) [25]	Check for associations and dose-response relationships between current exposure and cumulative exposure to night work and atherosclerosis	Interviews (including job data), blood sampling (fibrinogen and CRP) and clinical examinations (AS, BP and AH).	PWV (Digital photoplethysmography—Pulse Trace PCA 2™, Micro Medical Ltd., currently CareFusion)	EG = Night shift worker and CG = day worker
Skogstad et al. (2019) [26]	To present the initial results of a cohort of workers in industrial shifts.	Questionnaires, BP, HR, VO2max Test, blood analyses (lipids, HbA1c, CRP), exposure to toxins, AS (CBP, AP, AIx, PWV) and USCA	PWV (SphygmoCor XCEL^®^—AtCor Medical Pty Ltd., Sydney, Australia)	EG = shift worker, including night work and CG = day worker
Sugiura et al. (2019) [29]	To analyze how shift work and lifestyle habits interfere with the accumulation of visceral fat and the presence of atherosclerosis	Anthropometric data, BP, blood samples (lipids, creatinine, FBG, HbA1c), AS (CAVI e IMT) e VFA (CT).	CAVI (Vasera VS-1000 automatic system—Fukuda Denshi, Tokyo, Japan)	EG = Shift workers and CG= fixed daytime workers
Hannemann et al. (2020) [32]	To assess whether timed physical exercise interferes with glucose metabolism, circadian rhythms, and 24 h blood pressure in shift workers.	Questionaries, anthropometric data, job data, actigraphy, effects of timed physical, exercise performance, glucose tolerance, diurnal rhythms of melatonin and cortisol, and 24 h BP, AS, HOMA-IR and QUICKI	24 h-PWV (BPLab^®^—OOO Petr Telegin, Nizhny Novgorod, Russia)	IG = night workers with exercise and CG = night workers without exercise
Mamen et al. (2020) [27]	To examine whether high-intensity PA would modify the risk of CVD in the studied group.	Questionnaire, AP, BP, HR, CBP, AIx, PP, PWV, HbA1c, HDL, LDL, CHOL, CRP, VO2max, PAI	PWV (SphygmoCor XCEL^®^—AtCor	IG = ≥10 training sessions and CG = 0 ou < 10 training sessions
Schäfer et al. (2020) [30]	To assess whether timed aerobic exercise before night work reduces CVR and AS	35 min of HIIT/12 weeks, anthropometric data, glucose metabolism, lipid profile, exercise capacity and PWV.	PWV (Mobil-o-Graph^®^—NG (IEM, Stolberg, Germany)	IG = 35min HIIT before each NW and CG = no training
Hegewald et al. (2021) [31]	To examine whether WLC can impact AH incidence and cardiovascular health	Questionnaire (including COPSOQ), WLC, AH, Stiffness Index.	Stiffness Index (Pulse Trace PCA2 device—Micro Medical imited/Carefusion)	2 subsamples: (1) included AH and AS, and (2) excluded CVD (MI, stroke, AF, PAD, CAD, and CHF).
Matre et al. (2022) [24]	To investigate associations between sleep duration, number of awakenings, BP and AS in shift workers.	BP, HR, PWV, actigraphy	PWV (SphygmoCor XCEL^®^—Atcor Medical, New South Wales, Australia)	Analyzed sleep duration and number of awakenings according to: diary and actigraphy

BaPWV = brachial–ankle pulse wave velocity, BP = blood pressure, EG = exposure group, CG = control group, BMI = body mass index, AS = arterial stiffness, CW = clockwise, CC = counterclockwise, PWV = pulse wave velocity, CVR = cardiovascular risk, FBG = fasting blood glucose, CHOL = total cholesterol, AH = arterial hypertension, CAVI = cardio-ankle vascular index, VO2max = maximal oxygen uptake, HbA1c = glycosylated hemoglobin, CRP = C-reactive protein, CBP = central blood pressure, AP = augmentation pressure, Aix = augmentation index, USCA = ultrasound of the carotid arteries, IMT = intima media thickness, VFA = visceral fat area, CT = computed tomography, HOMA-IR = homeostasis model assessment of insulin resistance, QUICKI = quantitative insulin sensitivity check index, IG = intervention group, PA = physical activity, CVD = cardiovascular disease, HR = Heart Rate, PP = pulse pressure, HDL = high-density lipoprotein cholesterol, LDL = low-density lipoprotein cholesterol, PAI = personalized activity intelligence, HIIT = high-intensity interval training, NW = night work, WLC = work–life conflicts, COPSOQ = Copenhagen Psychosocial Questionnaire, MI = myocardial infarct, AF = atrial fibrillation, PAD = peripheral arterial disease, CAD = coronary artery disease, CHF = chronic heart failure.

**Table 5:** Systematization of main results, confounder, covariate selection, and limitations of the studies.

Author and Year	Main Results	Considered Confounder Factors	Limitations
Chen et al. (2010) [23]	BaPWV increased by 3.6 cm/s for per 1-year increment in years of shift driving	Age, education level, smoking, alcohol, tea and coffee consumption, PA, sleep time, WC, BP, BMI, CHOL, HDL, LDL, plasma glucose and insulin.	Working hours starting at different times. Noise and motor exhaust are possible CVD. Selecting healthy workers can have biased results Small sample limited statistical ability to detect risk.
Kantermann et al. (2013) [21]	There was no significant difference in PWV between shift-rotations (CW, CC and DW)	Analyzes involving PWV were adjusted for age, BP, HR, BMI, WHR and smoking as covariates.	The small sample may have influenced the lack of statistical difference in PWV between the two shift groups
Chou et al. (2015) [28]	The sleep duration < 6 h and weekly work hours > 60 h were significantly associated with increased risk of arteriosclerosis	Adjustments for age, gender, education, medical profession and CVR (smoking, BMI, HR, BP, FBG and CHOL) were included in the analysis model.	Lack of validation of the use of the BaPWV formula for the Taiwanese population. Included only medical staff. The sample size was modest, and the study design weakens the causal relationship.
Jankowiak et al. (2016) [25]	Night SW with >660 NS within the last 10 years increased AS (0.33 m/s), with 4% flow velocity increase compared to non-night workers.	Covariates included in the different regression models: basic confounders (age and gender), current occupational exposures, lifestyle factors (smoking, alcohol, WHR), Socioeconomic status and dispositional factors (menopause status, family history of MI or stroke).	The analyzes were of a transversal cut. There was no precise data on types of turns and direction of rotation. The number of valid measurements of vascular function was limited. Physical activity cannot be considered an adjustment.
Skogstad et al. (2019) [26]	Longer time (years) of SW was associated with increased IMT and high CRP.	Adjustments for age, gender and smoking were used in the analysis model.	Cross-sectional design, possible selection bias (workers) and CVD-related covariates (unhealthy eating habits and alcohol intake) were not considered.
Sugiura et al. (2019) [29]	VFA, CAVI, and IMT values were significantly greater in fixed daytime workers than in shift workers	Analyzes were performed with different models adjusted for: age, systolic BP, HDL, LDL, TGL, creatinine, VFA, CAVI, CCA and IMT in different ways.	The study design was cross-sectional. The background of the participants was heterogeneous. The categorizations considered only two groups
Hannemann et al. (2020) [32]	Timed exercise sessions before the NS have no significant effect on glucose tolerance, 24 h BP, and circadian rhythms of melatonin and cortisol.	Not reported	Small number of participants. Adoption of low-intensity exercise (weaker zeitgeber). Light PA and food intake were not controlled. Baseline fitness level was better in IG workers.
Mamen et al. (2020) [27]	Short training sessions with 4 min of high-intensity PA, 3 times/week/8 weeks among rotating SW reduced some CVD risk (BP, HbA1c).	Not reported	Misclassification of exercise level. Self-reported activities can be biased, especially low-intensity training activity.
Schäfer et al. (2020) [30]	12 weeks of HIIT within 2 h before NS work improves physical exercise capacity and AS (reduction of −0.1 ± 1.1m/s)	Not reported	Not reported
Hegewald et al. (2021) [31]	There was no association between WLC and incident hypertension or increased AS, but results stratified by gender resulted in a hazard ratio of 1.47 (95% CI 0.54–3.98) for incident CVD among women.	Depending on the model: sex, age, socioeconomic level, WLC (management position, NS and working hours/week), factors of private life, smoking, alcohol abuse and WHR.	Few incidents CVD were observed in the first five years among women. Selection bias may have occurred (healthy worker). They could not estimate the effect of private life roles interfering with work on cardiovascular health.
Matre et al. (2022) [24]	Shorter sleep duration was associated with higher BP and partly with higher PWV, indicating an increased risk of CVD with reduced sleep	Analyzes involving PWV were adjusted for gender and age	Sample size. Data not representative of the population. Sleep duration calculated by an actigraphy not validated against polysomnography. Wide divergence between actigraphy and diary data.

BaPWV = brachial–ankle pulse wave velocity, PA = physical activity, WC = waist circumference, BP = blood pressure, BMI = body mass index, CHOL = total cholesterol, HDL = high-density lipoprotein cholesterol, LDL = low-density lipoprotein cholesterol, PWV = pulse wave velocity, HR = heart rate, WHR = waist-to-hip ratio, CVR = cardiovascular risk, FBG = fasting blood glucose, MI = myocardial infarct, SW = shift workers, DW = day workers, CVD = cardiovascular disease, IMT = intima media thickness, TGL = triglyceride, VFA = visceral fat area, CAVI = cardio-ankle vascular index, CCA = common carotid artery, IG = intervention group, CG = control group, NS = night shift, WLC = work–life conflicts.

## 4. Discussion

According to the studies retrieved in this review, there is no uniformity in the results observed for early arterial stiffness as a predictor of vascular aging in shift workers. The studies reviewed differed in terms of study design, follow-up time, method of assessing arterial stiffness, exposure to shift work and night work, as well as observed results. Some studies reported that factors such as short sleep duration, BMI, systolic and diastolic blood pressure, fasting blood glucose, cholesterol, and smoking were associated with the risk of early arterial stiffness [24,28].

A few studies found no direct association between work shifts and arterial stiffness [21,30,32], others reported higher levels of arterial stiffness in day workers [26,29] whereas some studies showed that this indicator of arteriosclerosis was higher in shift workers [23,25]. These contradictory findings are surprising since there is evidence that shift work is associated with several health problems, including effects deleterious effects on the cardiovascular and metabolic systems [33,34,35,36,37,38,39,40].

The findings of Skogstad et al. [26] and Sugiura et al. [29] may have occurred due to the limitations highlighted in the studies in relation to the heterogeneity of the sample and possible selection bias and not considering covariates related to CVD (unhealthy eating habits and alcohol intake). In addition, the day workers studied by Skogstad et al. [26] were mostly older and with higher BMI when compared to night workers. A systematic review with meta-analysis, for instance, found a 17% higher risk of cardiovascular events, and an almost 20% higher risk of cardiovascular disease mortality, in shift workers compared to day workers [32].

Although the association between shift work and cardiovascular diseases is well described in the literature, with the work shift being recognized as an independent factor for cardiovascular diseases, the pathophysiological mechanisms involved are still not fully elucidated [30]. A possible explanation for this relationship is that the reduced synthesis of melatonin in shift workers can promote increased pressure and thrombus formation, since when the hormone is released in the ideal concentration, it could contribute to reducing blood pressure and increasing of blood fluidity [41,42]. Evidence indicates that reduced sleep time alters physiological processes in the body, including changes in heart rate [43], changes in blood clotting [44,45] dysregulation of immunity [46], mental disorders [47], and risk of cardiometabolic diseases [48,49,50].

Of the studies exploring the association between work shifts and early arterial stiffness, two identified an association between the time of exposure to work shifts, in the last 10 years, and early arterial stiffness [23,25]. Moreover, the study found a positive relationship between shift work and arterial stiffness with 3% higher levels when associated with a cumulative number in the last 10 years of night shifts among the workers analyzed. When analyzing cumulative work shifts and overall years worked, the association with increased arterial stiffness was even greater, reaching 4% [25]. In line with these findings, a metanalytic study found a 7.1% higher risk of CVD incidence for every five years of night work [51]. Another systematic review with meta-analysis, based on prospective observational studies, indicated approximately 40% higher risk of coronary heart disease in workers engaged in long working hours [52]. In fact, the risk of CVD in shift workers has previously been reported in the literature.

The study evaluating whether intentional exercise before night shifts could improve the cardiometabolic profile of workers observed that 24 h blood pressure and PWV values remained practically unchanged before and after the period of intervention with exercise [32]. In this same line of research, another author assessed the impact of high-intensity exercise two hours before night work and observed subtle changes in blood pressure and PWV values, but these were not significant when compared to the control group throughout the study [30].

Studies about physical exercise and PWV among night workers might be interesting to investigate whether the activation of the circulatory and metabolic systems alleviates the circadian disruption observed in this population (in addition to glucose regulation and blood pressure regulation) [53].

One of the studies found in the review, investigating the impact of work–life conflicts on workers’ health, with five years of follow-up, observed no association between worse work–life conflict and hypertension or increased arterial stiffness. However, gender stratification showed an increased risk for women [31]. López-Soto et al. [54] highlighted that women, when working in shifts, have more sleep disturbances than men, especially when they need to perform housework and care for small children, as they tend to restrict their rest time to perform the demands of home care. One of the most accepted mechanisms for the genesis of cardiovascular changes in shift workers is psychological stress. It is suggested that the stress triggered by circadian disruption, caused by exposure to shift work and routine instability, promotes hypersecretion of cortisol and catecholamines, contributing to changes in physiology [9,10].

Although the association between shift work and arterial stiffness has not been fully elucidated by the studies included in this review, findings showed there are risk factors involved in the etiology of arterial stiffness. Risk factors previously associated with shift work include high levels of cholesterol, uric acid, glucose, and potassium. Another aspect to be considered is that night shift work has been associated with an increased risk of chronic systemic inflammation, which potentiates cardiac risks [51,52].

The metabolic and physiological changes in circadian disruption can cause changes in lipid metabolism, represented by an increase in triglycerides, an increase in total cholesterol, and a reduction in high-density lipoprotein. In addition to the lack of control of the mechanisms of the hypothalamic–pituitary axis of hunger and satiety, triggered by increased resistance to leptin and insulin, predisposing to obesity [55,56]. These conditions are known to be involved in the etiology of health problems and can be potentiated by other predisposing factors such as family history, hypertension, diabetes, smoking, sedentary lifestyle, and inadequate diet [10].

Epidemiological studies have shown that there is an increased risk of cardiovascular diseases in individuals who work in shifts [56,57]. It has been suggested that this risk may be due to the stress generated by years of exposure to irregular shifts or due to the accumulation of classic cardiovascular risk factors that are more present in shift workers, including: smoking, low physical activity, overweight or dyslipidemias (triglycerides, total cholesterol, low-density lipoproteins—LDL above the normal limits and/or high-density lipoproteins—HDL below the normal value) [55].

Matre et al. [24] and Chou et al. [23] identified an association between short sleep duration and arterial stiffness. It is suggested that this occurs because sleep deprivation can cause biological outcomes such as increased inflammation, and hormonal and metabolic changes, which disorganize the collagen and elastin fibers of the vascular wall [58] and may increase arterial stiffness [59]. The higher prevalence of CVD in individuals with sleep deprivation, which occurs during work shifts, may be associated with changes in circadian patterns, reduced vascular elasticity, and, consequently, increased arterial stiffness [5,60]. A systematic review with meta-analysis, for instance, found a 17% higher risk of cardiovascular events, and an almost 20% higher risk of cardiovascular disease mortality, in shift workers compared to day workers [61].

Several hypotheses have been suggested to explain the higher incidence and prevalence of CVD in shift workers. One of these hypotheses holds that night shift workers are less likely to adopt a healthy lifestyle [8]. The association between inadequate lifestyle and chronic sleep restriction may render professionals who work at night more exposed to endothelial dysfunction, loss of arterial elasticity, and vascular stiffness, which are early markers of CVD [62]. Another assumption underlying the increased risk of CVD seen in shift workers, especially night workers, is that exposure to work at night causes circadian disruption in blood pressure control mechanisms [11]. The repercussions of shift work on blood pressure can trigger arterial stiffness and possibly culminate in premature vascular aging. It is noteworthy that arterial stiffness is also naturally influenced by the age, genetics, and gender of the individual, being an early predictor of cardiovascular disease and target organ damage [62].

Findings on shift work and arterial stiffness remain heterogeneous, precluding any robust conclusions. Yet, the present review complements a growing body of evidence on the impact of shift work and the risk of atherosclerosis, which potentiates the emergence of cardiovascular disease. In addition, shift work, as cited in the articles reviewed, exposes workers to shorter sleep duration, artificial light at night, and both physical and mental stress. All these changes can misalign circadian rhythms, alter blood pressure regulatory mechanisms, and increase the risk of metabolic disorders, chronic inflammation, arterial stiffness, and cardiovascular outcomes. Occupations, different forms of organization of shift work, including night work or otherwise, weekly exposure dose, and cumulative exposure time over the years of night work are factors, which also play a role.

Due to the diversity of study designs and the conflicting results of the studies included in this review, it cannot be said that there is an association between shift work and arterial stiffness. There is a need for more studies with well-controlled, longitudinal methods and with samples that allow population inference so that robust evidence can be produced.

## 5. Conclusions

The studies included in this review evaluated arterial stiffness using different methods, obtained conflicting results, and do not allow us to state that shift work is an independent predictor of arterial stiffness. However, considering the papers just mentioned, there are many studies that show that different factors (sleep deprivation, circadian disruption, and lack of adoption of a healthy lifestyle) are capable to increment the prevalence of CVD. Further studies with a longitudinal design and control of confounding factors are needed to establish whether there is a causal relationship between shift work and arterial stiffness.

## Figures and Tables

**Table 1:** Research question devised using PICO strategy.

Item	Definition
Population	Adults, aged > 18 years
Intervention/Exposure	Night shift work
Control	Non-shift workers and day workers
Outcomes	Early arterial stiffness as a possible trigger of cardiovascular risk

**Table 2:** Databases, keywords, and criteria for searches.

Databases Searched	Key Words (MeSH Terms) to Create Search Strings
Cochrane	Shift work; shift worker; schedule shift work.
Embase	Night work; night shift work.
PubMed	Arteriosclerosis; atherosclerotic risk.
Scielo	Stiffness, vascular; vascular stiffnesses; arterial stiffness; arterial stiffnesses; stiffness, aortic.
Scopus	Pulse wave velocity; pulse wave velocities.
No filters were used	Strings were created according to each database using the Boolean operators OR and AND.
**Selection Criteria for Inclusion Based on Title, Abstract and Eligibility Stage**	
**Inclusion:**	**Exclusion:**
Quantitative studies related to shift work, including night shifts and arterial stiffness.	Experimental studies.
Original and research articles, longitudinal studies, cohort studies, and case–control studies.	Review articles not including quantitative data.
Available online with free access and as full text.	

## Data Availability

Not applicable.
